# Annotations

**Published:** 1906-03-10

**Authors:** 


### ANNOTATIONS.

### The Vagrant.

We have often had occasion to refer to the evils of vagrancy and to lament because the methods which up to the present have been used to combat this social evil are utterly illogical and inadequate. To-day the tramp is rather encouraged than hindered in his evil waywardness; and the restrictions and penalties that may be imposed upon him are by no means of such a nature as to induce him to relinquish his wandering habits and face out the dull monotony of steady work. For this reason we welcome the report of the Departmental Committee on Vagrancy, which has just been published; for their recommendations are drastic and the reforms they advocate are radical. The Committee put forward a scheme whose main designs are to transfer the responsibility for dealing with vagrants from the Guardians to the police, to help the *bona fide* wayfarer, and to provide a means of detaining the habitual vagrant under reformatory influences. In future, all ordinary casuals will be detained for two nights, and will have to do a good day's work in the interval, and they will not be supplied with a ration to carry away with them from the casual ward, but will have to apply for it at the nearest police station; an arrangement which serves the two objects of removing an excuse for begging and of bringing the individual concerned in close contact with the police. We have not space to mention all the recommendations contained in the report, but it is fair to say that if they ever become law they will go a long way towards eliminating the dirty, disease-carrying, expensive nuisance which the habitual vagrant is admitted to be. The important part played by tramps in the dissemination of small-pox during the recent epidemic in this country was more than once demonstrated beyond a doubt, and it is certain that they are fruitful agents also in the spread of enteric fever and other infectious maladies. By enforcing such regulations as the Departmental Committee recommend, this source of danger to the public will be to a considerable extent eliminated.

### Underfed School Children.

The Education (Provision of Meals) Bill has passed its second reading and been referred to a select committee. Some such measure results as a natural sequence of the premiss whose acceptance gave free education to the children of the country. Whatever be the political views of the profession upon a measure frankly socialistic like this, it is, as Sir William Collins observed in the course of the debate, impossible to traverse the proposition that "to ask teachers to instil knowledge into the ill-nourished and anæmic brains of underfed children was to ask them to perform, at best, a stupid miracle." Fortunately perhaps for the profession, the political aspect of the problem is outside the purview of medicine. We have to deal with facts as they are, and to regulate our public and practical, as opposed to our private and academic, attitude accordingly. Considered in this light, the proposition laid down by Sir William Collins must be held by all of us to be incontrovertible. More than this, the same speaker laid stress upon the fact that among other unfavourable conditions which precluded children from deriving full benefit from their education was an insufficiency of clothing. It would be no difficult matter to collect a large body of evidence to show that this is almost a more important matter than the other. Those who have any knowledge of young children know well that warmth is essential to their welfare, and that a very fruitful source of nutritional disorders among the children of the poor is a lack of sufficiently warm clothing, especially for the abdomen and lower extremities. Such a lack, by impairing the digestion, as it undoubtedly will, reduces the value of such food-stuffs as are ingested, and thus completes a vicious circle whose expression is chronic intestinal dyspepsia, if not maladies more grave. If it were not that logic is no necessary part of government, we should not wait long for an Education (Provision of Clothing) Bill: indeed, we may soon get it in spite of that circumstance, and it will be hard to deny it some sound justification.

### Tea as a Disease Producer.

Attacks on licensed victuallers are common and often unfair. At Preston they have been pursued with such bitterness, and with such marked disregard of the elementary principles of justice, that a well-known medical man has been moved to try and turn the tables on the total abstainers, or at least the considerable portion of them who are inveterate consumers of tea. He asserts his belief, which is founded upon a long professional experience, that the moderate and reasonable use of beer as a beverage is less harmful than the same use of tea, and that immoderate tea-drinking causes more pain, suffering, ill-health, and nervous breakdown than the excessive consumption of beer. Admitting that if a person imbibes too much beer the effects are gross and palpable, he contends that the results of consuming too much tea are quite as bad, or even worse, but that as they are insidious and not generally apparent, they are put down to some other cause; and he declares that amongst the evil effects produced by tea are anæmia, chronic gastritis, dyspepsia, and emaciation, while it also lays the foundation of gastric ulcer, causes irritability of the nerves, and a whole host of nervous disorders. All this is more or less true if it be not new. But the enumeration of the evils of excessive tea-drinking is followed by the novel suggestion that any one desirous of amassing a fortune should make up pills of innocent materials and in the directions for their use stipulate that the patient should, while taking the remedy, refrain from the use of tea. The author of the suggestion thinks that such pills would have an enormous sale, do a vast amount of good, and relieve a great amount of suffering. They might, if the purchasers observed the directions. We do not believe that they would, and even our conviction that the abuse of tea, like the abuse of alcohol, is responsible for much physical disability and premature degeneration, does not impel us to advocate the introduction of another mysterious pill.

